# Integrated transcriptomic analysis reveals hub genes involved in diagnosis and prognosis of pancreatic cancer

**DOI:** 10.1186/s10020-019-0113-2

**Published:** 2019-11-09

**Authors:** Yang-Yang Zhou, Li-Ping Chen, Yi Zhang, Sun-Kuan Hu, Zhao-Jun Dong, Ming Wu, Qiu-Xiang Chen, Zhi-Zhi Zhuang, Xiao-Jing Du

**Affiliations:** 1 Department of Rheumatology and Immunology, The Second Affiliated Hospital and Yuying Children’s Hospital of Wenzhou Medical University, Wenzhou, 325000 Zhejiang Province China; 2 Chemical Biology Research Center, College of Pharmaceutical Sciences, Wenzhou Medical University, Wenzhou, 325000 Zhejiang China; 3 Department of Gastroenterology, The First Affiliated Hospital of Wenzhou Medical University, Wenzhou, 325000 Zhejiang Province China; 4 Department of Ultrasound, The First Affiliated Hospital of Wenzhou Medical University, Wenzhou, 325000 Zhejiang Province China

**Keywords:** Pancreatic cancer, Diagnosis, Prognosis, Integrated transcriptomic analysis

## Abstract

**Background:**

The hunt for the molecular markers with specificity and sensitivity has been a hot area for the tumor treatment. Due to the poor diagnosis and prognosis of pancreatic cancer (PC), the excision rate is often low, which makes it more urgent to find the ideal tumor markers.

**Methods:**

Robust Rank Aggreg (RRA) methods was firstly applied to identify the differentially expressed genes (DEGs) between PC tissues and normal tissues from GSE28735, GSE15471, GSE16515, and GSE101448. Among these DEGs, the highly correlated genes were clustered using WGCNA analysis. The co-expression networks and molecular complex detection (MCODE) Cytoscape app were then performed to find the sub-clusters and confirm 35 candidate genes. For these genes, least absolute shrinkage and selection operator (lasso) regression model was applied and validated to build a diagnostic risk score model. Cox proportional hazard regression analysis was used and validated to build a prognostic model.

**Results:**

Based on integrated transcriptomic analysis, we identified a 19 gene module (*SYCN*, *PNLIPRP1*, *CAP2*, *GNMT*, *MAT1A*, *ABAT*, *GPT2*, *ADHFE1*, *PHGDH*, *PSAT1*, *ERP27*, *PDIA2*, *MT1H*, *COMP*, *COL5A2*, *FN1*, *COL1A2*, *FAP* and *POSTN*) as a specific predictive signature for the diagnosis of PC. Based on the two consideration, accuracy and feasibility, we simplified the diagnostic risk model as a four-gene model: 0.3034*log_2_(*MAT1A*)-0.1526*log_2_(*MT1H*) + 0.4645*log_2_(*FN1*) -0.2244*log_2_(*FAP*), log_2_(gene count). Besides, a four-hub gene module was also identified as prognostic model = − 1.400*log_2_(*CEL*) + 1.321*log_2_(*CPA1*) + 0.454*log_2_(*POSTN*) + 1.011*log_2_(*PM20D1*), log_2_(gene count).

**Conclusion:**

Integrated transcriptomic analysis identifies two four-hub gene modules as specific predictive signatures for the diagnosis and prognosis of PC, which may bring new sight for the clinical practice of PC.

## Introduction

Pancreatic cancer (PC) is a common malignant tumor of digestive system and ranks the fourth leading cause of cancer-related death worldwide (Kamisawa et al., 2016). The prognosis of PC is grim, with patients’ displaying the 5-year survival rate of only 8% (Siegel et al., 2016). The high mortality of PC patients mainly attributes to the inability to diagnose the disease early and the cancer being highly resistant to treatment (Ryan et al., 2014). Though recent advances in the diagnosis of PC have being evaluated, PC patients are often diagnosed at a advanced stage, due to non-specific clinical symptoms, the lack of truly effective conventional imageological examinations that will identify early stage, and the absence of specific and sensitive diagnostic biomarkers (Ryan et al., 2014). Hence, it is exceptionally urgent to establish novel diagnostic molecular markers for PC (Resovi et al., 2018; Tempero et al., 2013). In addition, a part of patients diagnosed at early stage also suffer a miserable ending, because of the high grade malignant of PC. It is also necessary to monitor patients at high risk for poor clinical outcome and identify novel prognostic molecular markers as early diagnostic biomarkers.

Technological development largely catalyzed our understanding of cancer genomics. Since the first publication of serial analysis of gene expression (SAGE) technique in 1995 (Velculescu et al., 1995), high-throughput gene expression analysis has revolutionized cancer genetics over the last 15 years (Chibon, 2013). A comprehensive genetic analysis of 24 pancreatic cancers sequenced the coding region of 20, 661 genes and indicated the genetic landscape of PC (Jones et al., 2008). Four frequently mutated genes have been identified in PC, including *CDKN2A* (*p16*), *SMAD4* (*DPC4*), and *TP53* tumor suppressor genes and *KRAS* oncogene (Jones et al., 2008). Several candidate cancer genes that alter at low frequency are also identified such as *MLL3* and *ARID1A* (Jones et al., 2008; Balakrishnan et al., 2007). These four frequently mutated genes are well recognized as contributing to the carcinogenesis of PC and regarded as the “driver” genes for this tumor (Iacobuzio-Donahue, 2012), while the diagnostic value of these altered genes for PC need to be further estimated. *CA19–9* is the common applied serologic marker for the diagnosis of PC in clinic (Ballehaninna and Chamberlain, 2012). However, *CA19–9* has limited performance in detecting early-stage disease (Ballehaninna and Chamberlain, 2012). Hereby, specific and sensitive diagnostic gene models have always been pursued by cancer researchers. But, there has been few gene diagnostic model with high sensitivity and specificity for PC hitherto. The similar predicament has also existed in the study of prognostic biomarkers of PC. Though numerous genes, as *ACTN4*, *LMO2*, *p16INK4a*, have been reported to be involved in the prognosis of PC (Watanabe et al., 2015; Nakata et al., 2009; Gerdes et al., 2002), none of them have been applied in clinic.

In the recent years, gene expression data from the public database, such as the Cancer Genome Atlas Cancer Genome (TCGA), Gene Expression Omnibus (GEO), offer available information with respect to the molecular mechanism and variety of multiple carcinomas, and are of great value to the diagnosis, prediction of progression in these disease (Chibon, 2013). In this paper, Robust Rank Aggreg (RRA) methods were employed to identify the differentially expressed genes (DEGs) from four PC genome expression datasets. Then, bioinformatics method of weighted gene co-expression network analysis (WGCNA) was applied to identify the gene modules with importance. We next performed the co-expression networks and molecular complex detection (MCODE) of Cytoscape app to find the sub-clusters and confirm the hub genes. Finally, two prediction models, involved in the diagnosis and prognosis, were established.

## Materials and methods

### Collection of pancreatic cancer related genome expression datasets

All of the PC associated datasets were firstly downloaded from GEO (http://www.ncbi.nlm.nih.gov/geo/). A comprehensive assessment of each database was then performed with specific criteria. The selection criteria for this article are as follows: 1. one of the gene microarray technology or RNA-Seq technique must be included in genome expression profiling datasets; 2. DEGs between PC and normal tissues require to be detected in human samples and not in cell lines or other body fluid. Four databases, including GSE28735 (Zhang et al. 2012), GSE15471 (Badea et al., 2008), GSE16515 (Pei et al., 2009), GSE101448 (Klett et al., 2018) were selected as datasets for RRA analysis. GSE78229 (Wang et al., 2016) was selected as training dataset in the matter of prognosis (Table 1).

**Table 1:** Characteristics of gene microarray of this study

Reference	GEO	Platform	Normal	Tumor
Zhang et al	GSE28735	GPL6244	45	45
Badea et al	GSE15471	GPL570	39	39
Pei et al	GSE16515	GPL570	16	36
Klett et al	GSE101448	GPL10558	19	24
Wang et al	GSE78229	GPL6244	0	50

*GEO* Gene Expression Omnibus

### Datasets processing

After downloading series matrix files of GSE28735, GSE15471, GSE16515, GSE101448 from GEO, we normalized samples of each matrix files by “normalizeBetweenArrays” (Additional file 1: Figure S1) and identified the initial candidate genes of each dataset by the package “limma” of R (version 3.5.1, http://www.r-project.org/) (Ritchie et al., 2015), setting log_2_(Fold Change) ≥ 1, adjusted *P* < 0.05 as standard. The package “impute” was used to complete missing expression data. The DEGs were then identified by R package “Robust Rank Aggreg” and selected to construct a new data frame with log_2_(Fold Change) ≥ 1, adjusted *P* < 0.05. RRA method uses a probabilistic model for aggregation to monitor genes that are ranked consistently better than expected under null hypothesis of uncorrelated inputs and allocates a significance score for each gene (Kolde et al., 2012).

### WGCNA

WGCNA is a network biology method that is functioned to cluster the highly correlated genes and identify the co-expression modules. The highly correlated genes are used to construct correlation networks, which facilitate gene screening methods that can be used to identify candidate biomarkers (Langfelder and Horvath, 2008). The gene chip of GSE28735 was selected to identify the co-expression modules for having a relatively large number of samples and relatively detailed data of survival index. The cutHeight = 0.8 and minSize = 10 were applied to identify modules.

### Enrichment analysis

Gene Ontology (GO) analysis was conducted by the PANTHER classification system for the enrichment analyses (http://pantherdb.org/) (Mi et al., 2019). The statistical test was Fisher’s Exact and False Discovery Rate (FDR) < 0.05 was considered as statistically significant difference. The functional annotation of genes were reflected in cellular component, biological process, and molecular function, three major GO classifications (Ashburner et al., 2000; The Gene Ontology Consortium, 2017). Kyoto Encyclopedia of Genes and Genomes (KEGG) pathways (Kanehisa et al., 2017) and Reactome pathway (RP) (Fabregat et al., 2018) were performed to analyze related significant pathways. The enrichment analysis was visualized by Graphad Prism 5.0 (La Jolla, CA).

### Co-expression network and MCODE analysis

The STRING (https://string-db.org/) was served to identify the pairwise relationships of all genes (Szklarczyk et al., 2017). The co-expression networks of different modules were firstly constructed through the employment of STRING. The cut-off for confidence scores of interactions is 0.4. To further analyze the physical relationships among these distance-related genes, MCODE algorithm was used to select the clusters of the co-expression networks with the default settings: node score cutoff 0.2, K-core: 2 (Bader and Hogue, 2003). The sub-clusters were visualized by Cytoscape (version 3.6.0). The genes in sub-clusters were selected as candidate genes for diagnosis and prognosis analysis of PC.

### Construction and validation of diagnostic risk model

The gene expression profile of GSE28735 (*n* = 90) were served as training cohort to build least absolute shrinkage and selection operator (Lasso) regression model for diagnosis. Lasso regression is a kind of penalized regression method, which identifies regression coefficients for genes to shrink a weighted average of mean squared prediction error for cases (Zhao and Simon, 2010; Cai et al., 2018). The risk score model of Lasso regression was built by the package “LARS” of R (Xiao et al., 2015). To further determine the superiority of risk score model, we assessed the sensitivity and specificity of genes that formed risk model and the risk score model respectively, receiver operating characteristic (ROC) analysis was performed and the area under the curve (AUC) value was calculated to compare the prognostic performance. Besides, the gene expression profile of GSE16515 (*n* = 52) was used as validation cohort to verify the ability of diagnostic risk score model. Since the optimum model contained too many mRNAs to diagnose PC, we deleted mRNAs with low weight step by step and then rebuilt lasso regression model. We set AUC > 0.90 as the cut-off value to get the simplified diagnostic risk model with minimum quantity of gene count.

### Construction and validation of prognostic risk model

The gene expression profile of GSE78229 (*n* = 49) was served as training cohort to build prognostic model. Cox proportional hazard regression model was completed by SPSS (version 20.0). Additionally, the gene expression profile of GSE28735 (*n* = 41) and TCGA (https://cancergenome.nih.gov/) were used as validation cohort to verify the prognostic ability of risk score model. The results were displayed with hazard ratios (HRs) and coefficient with 95% confidence intervals (95%CI). The package “survminer” of R was applied to visualize the survival curves.

## Results

### Identification of the DEGs by integrated analysis

**Fig. 1:**
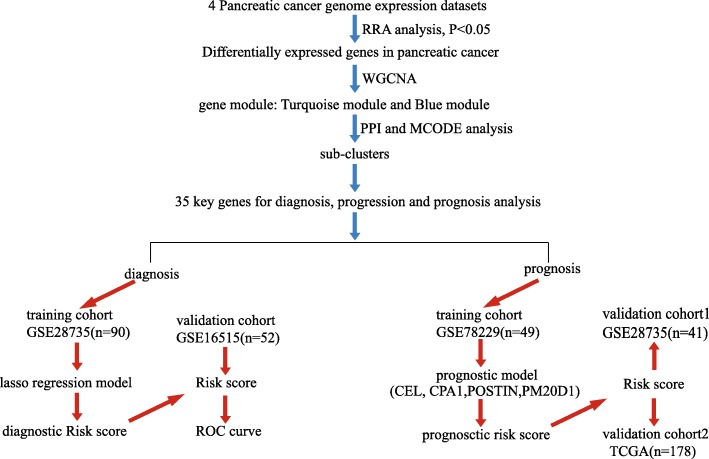
Flow diagram describing the design process of this study

**Fig. 2:**
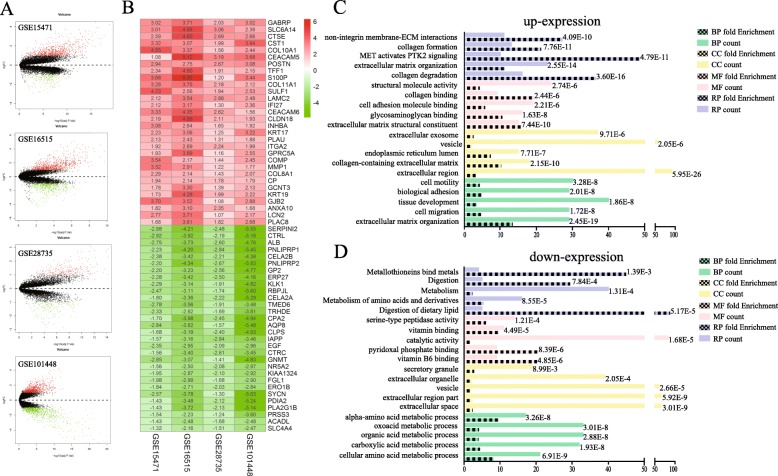
Identification of differentially expressed genes and their enrichment analysis. **a** The volcano plots of gene chips. **b** Heatmap displayed the log_2_(Fold change) of top 30 high expression genes and 30 low expression genes selected by Robust Rank Aggreg (RRA) methods from 4 independent gene chips. Each row represented the same mRNA from different gene chips and each column represented the same chip. The log_2_(Fold change) tendency of each mRNA was displayed in shade of red or green and the values of log_2_(Fold change) were marked within each box. Red represented the fold change of up-expressed genes and green represented down-expressed genes, respectively, compared to para-carcinoma tissues. **c-d** Gene ontology (GO) enrichment analysis and Reactome pathway enrichment analysis of high expression genes (**c**) and low expression genes (**d**). The vertical axis represented GO term or pathway, and the horizontal axis represented count of genes or fold enrichment. The column with black patches represented fold enrichment. The value of false discovery rate was shown at the end of each column. BP, biological process; CC, cellular component; MF, molecular function; RP, Reactome pathway

To describe the design process of this study, a flow diagram was indicated in Fig. 1. Before RRA analysis, differentially expressed genes were identified of each gene chip with log_2_(Fold Change) ≥ 1, adjusted *P* < 0.05 as standard (Fig. 2a). In total, there were 138 up-expressed genes and 165 down-expressed genes identified as DEGs with statistical significance through the integration of 4 datasets using RRA method. The top 30 of up-expressed genes and down-expressed genes were shown in Fig. 2b. GO enrichment analysis and Reactome pathway enrichment analysis of these up-expressed genes and down-expressed genes were then carried out and the results were shown in Fig. 2c and d. It was found that in these up-expressed genes, the top 3 enriched Reactome pathways were collagen degradation, extracellular matrix organization and PTK2 signaling pathway. As for biological process, cellular component, and molecular function, the DEGs mainly concentrated on the pathways of extracellular matrix organization, collagen degradation, vesicle, biological and metabolism. The above results indicated that these DEGs might be the key genes in pancreatic cancer.

### WGCNA and co-expression analysis

**Fig. 3:**
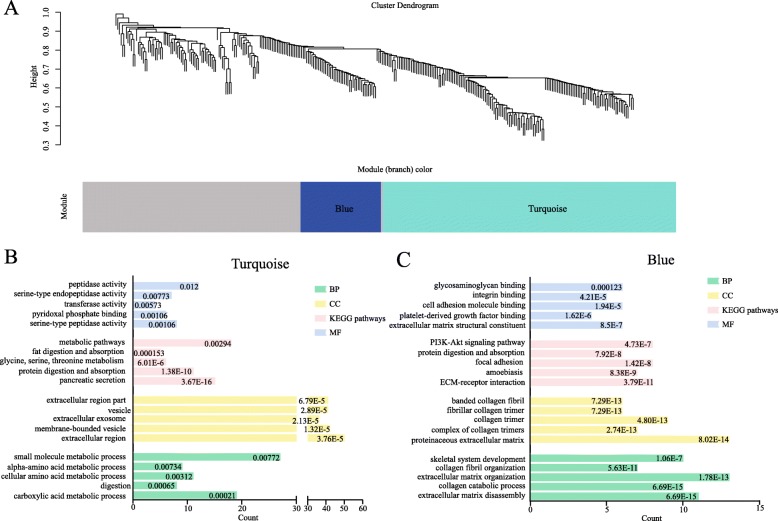
WGCNA analysis of the differentially expressed genes and Gene ontology enrichment analysis and KEGG pathway analysis of the functioned modules (**a**) Gene clustering and module identification was made by WGCNA analysis on the basis of the gene chip-GSE28735. Cluster dendrogram diaplayed the result of hierarchical clustering, and each line represented a gene. The colored column below the dendrogram represented module conducted by the static tree cutting method. The blue and turquoise color show different co-expression network modules for DEGs and grey module represents insignificant module. **b**-**c** Gene ontology enrichment analysis and KEGG pathway enrichment analysis of turquoise (**b**) and blue (**c**) module. The vertical axis represented GO term or pathway, and the horizontal axis represented count of genes. The value of false discovery rate was shown at the end of each column. BP, biological process; CC, cellular component; MF, molecular function; KEGG, Kyoto encyclopedia of genes and genomes; WGCNA, weighted gene co-expression network analysis

**Fig. 4:**
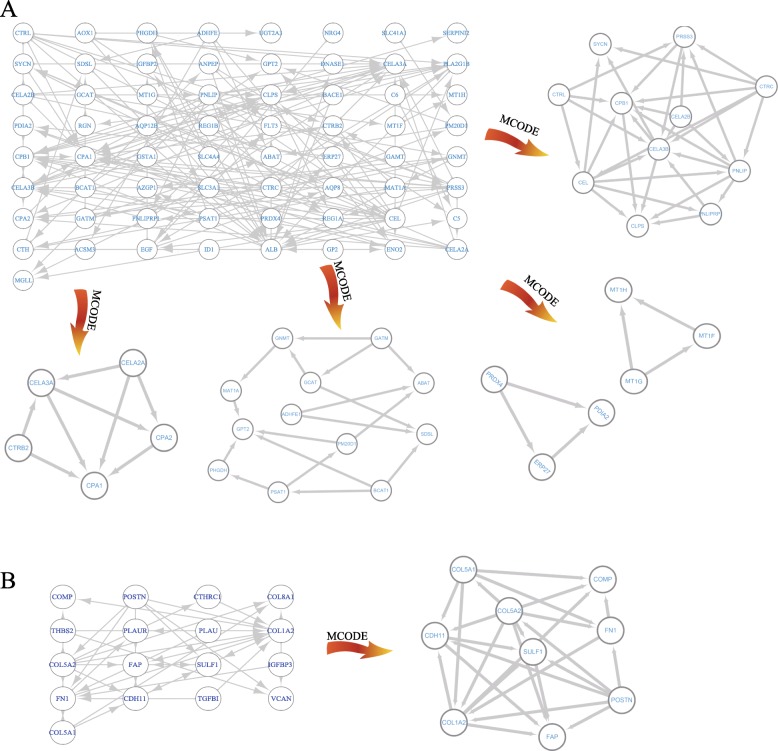
Visualization and identification of key genes form turquoise and blue module. **a** Co-expression network of turquoise module and sub-clusters that were highly interconnected regions in the network based on topology. **b** The co-expression network and sub-clusters of blue module

Based on the gene chip of GSE28735, WGCNA analysis was performed to cluster the highly correlated genes that mentioned above. These genes were mainly divided into three parts, of which blue and turquoise modules were considered as the most significant parts (Fig. 3a). As shown in Fig. 3b, in regard to KEGG pathway, turquoise module primarily focused on pancreatic secretion, protein digestion, glycine, serine threonine metabolism and fat digestion and absorption, while the blue modules involved in ECM-receptor interaction, focal adhesion, protein digestion and absorption and PI3K-Akt signaling pathway. To further identify the key genes from turquoise and blue module, co-expression networks of these two modules were constructed by STRING. Through the application of MCODE app by Cytoscape software, six sub-clusters were found and visualized that extracted from the turquoise and blue module (Fig. 4). The genes of sub-clusters were served as key genes for diagnosis and prognosis analysis of PC (Table 2).

**Table 2:** List of 35 key genes in four gene chips

Gene	GSE28735		GSE15471		GSE16515		GSE101448	
	logFC	adj P	logFC	adj P	logFC	adj P	logFC	adj P
CTRL	−2.19	2.22E-5	−2.92	8.42E-4	−3.92	7.73E-3	−5.18	2.7E-22
SYCN	− 1.30	1.94E-4	−2.57	7.54E-3	−3.78	2.59E-2	−5.63	8.31E-21
PRSS3	−1.24	5.67E-4	−1.54	3.08E-2	−2.22	3.85E-2	−3.60	7.79E-12
CTRC	−2.61	4.62E-5	−1.56	8.09E-2	−3.40	3.93E-2	−3.45	3.77E-10
CELA2B	−2.21	1.12E-4	−2.38	8.13E-3	−3.42	1.53E-2	−4.38	1.08E-23
CEL	−2.51	1.36E-4	−1.27	1.86E-1	−3.51	3.85E-2	−3.52	1.61E-10
PNLIPRP1	−2.83	3.73E-5	−2.23	1.32E-2	−4.20	5.89E-3	−5.45	6.64E-14
CPA1	−1.87	1.39E-3	−1.27	2.22E-1	−3.33	7.09E-2	−4.96	3.24E-13
CPA2	−2.45	2.78E-4	−1.70	5.76E-2	−3.98	1.26E-2	−4.94	1.90E-14
GNMT	−1.41	1.77E-4	−2.85	1.03E-6	−3.07	3.94E-4	−4.83	5.59E-19
GATM	−1.46	7.98E-4	−0.93	8.45E-6	−2.72	1.23E-3	−3.08	1.52E-14
MAT1A	−0.80	2.28E-4	−1.50	9.31E-08	−1.41	6.06E-3	−1.27	8.05E-5
GCAT	−0.57	6.11E-4	−1.55	6.87E-7	−1.46	6.00E-	−2.48	1.11E-6
ABAT	−1.12	4.47E-7	−1.55	1.05E-6	−2.19	6.19E-5	−1.84	2.21E-14
GPT2	−0.87	1.20E-3	−1.43	5.99E-6	−1.48	1.56E-2	−2.13	1.86E-13
ADHFE1	−1.09	3.11E-8	−0.75	3.05E-8	−1.31	8.46E-7	−2.21	9.28E-11
PM20D1	−1.40	7.49E-5	−2.48	2.43E-7	−2.01	4.71E-3	−0.70	5.67E-3
PHGDH	−0.88	6.05E-5	−1.08	8.35E-7	−1.15	6.47E-3	−1.23	1.03E-6
PSAT1	−0.98	1.82E-3	−2.29	5.09E-8	−1.93	2.16E-3	−1.12	2.48E-5
BCAT1	−0.52	2.79E-2	−1.07	6.20E-3	−1.06	5.80E-2	−1.62	2.67E-8
PRDX4	−0.64	4.42E-4	−1.22	2.09E-6	−1.35	2.82E-4	−1.08	4.64E-2
ERP27	−2.50	2.31E-5	−2.28	2.08E-3	−3.42	5.15E-3	−4.16	2.57E-15
PDIA2	−2.12	7.91E-6	−1.43	1.11E-6	−3.48	1.06E-3	−5.23	1.38E-21
MT1H	−0.70	1.20E-5	−1.02	1.17E-5	−1.87	3.05E-6	−3.53	1.39E-17
MT1F	−1.00	4.95E-5	−1.23	3.68E-6	−1.86	8.16E-6	−2.32	1.79E-7
MT1G	−1.15	4.34E-5	−1.65	7.28E-8	−2.15	4.75E-6	−2.53	3.04E-10
COL5A1	0.77	4.83E-6	3.22	3.83E-15	1.81	1.71E-3	1.99	3.02E-8
COMP	1.44	1.47E-6	3.54	1.28E-14	2.17	5.19E-3	2.45	1.59E-7
CDH11	1.43	2.19E-7	2.55	8.28E-14	1.72	2.56E-3	2.20	5.03E-4
COL5A2	1.33	9.57E-6	3.54	1.69E-15	1.71	1.43E-3	1.74	8.66E-6
FN1	2.31	2.65E-10	2.80	2.66E-17	2.30	5.10E-6	1.45	4.92E-9
SULF1	1.94	9.20E-9	4.23	1.17E-19	2.56	6.86E-5	2.53	2.47E-6
COL1A2	1.58	7.27E-6	3.43	1.18E-16	1.68	7.98E-4	1.57	7.13E-11
FAP	1.44	1.44E-5	3.26	3.72E-16	1.84	4.73E-3	2.23	1.03E-6
POSTN	2.67	1.13E-10	2.94	1.58E-14	2.75	6.46E-5	3.08	1.76E-12

logFC:log_2_(fold change) (tumor compared to normal tissue); *adj P:* adjusted *P* value

### Construction and validation of diagnostic risk model

**Fig. 5:**
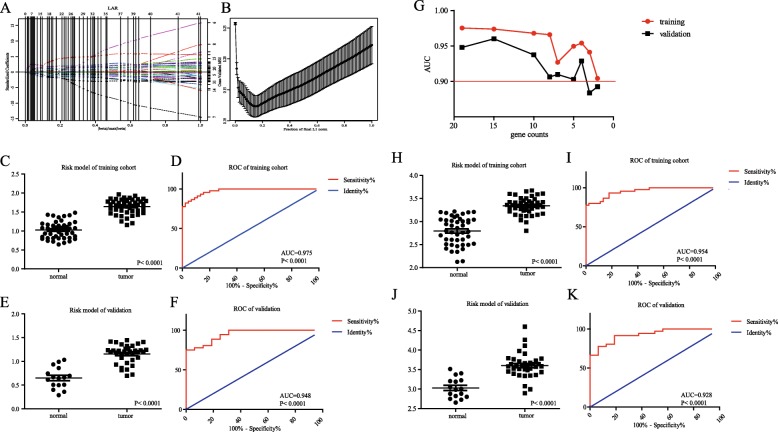
Construction of risk model for diagnosis of pancreatic carcinoma and ROC curve. **a** The solution paths of lasso regression model. The numbers on the right represented which variable each path corresponds to. Horizontal axis represented |beta|/max|beta| and vertical lines showed the event times for easy comparison between various solution paths. **b** The relationship between cross-validated mean square error (CV MSE) and model size. Horizontal axis represented fraction of final L1 norm, which referred to the ratio of the L1 norm of the coeffcient vector relative to the norm at the full least squares solution for the model with the maximum steps used. **c** The distribution of risk score of normal and tumor group of training set-GSE28735. **d** ROC curve of risk score for differentiating tumor from normal of training set. **e** The distribution of risk score of normal and tumor group of validation set-GSE16515. **f** ROC curve of risk model for differentiating tumor from normal of validation set. **g** The relation between gene counts that involve in diagnostic risk model and AUC of training cohort and validation cohort. **h** The distribution of risk score of normal and tumor group of training set-GSE28735 for simplified diagnostic risk model. **i** ROC curve of risk score for simplified diagnostic risk model of training set. **j** The distribution of risk score of normal and tumor group of validation set-GSE16515 for simplified diagnostic risk model. **k** ROC curve of risk score for simplified diagnostic risk model of validation set. AUC, area under the curve; ROC curve, Receiver operating characteristic curve

We constructed lasso regression analysis using these key genes by R language. As a result, optimal diagnostic risk model = 0.0504*log_2_(*SYCN*)-0.0492*log_2_(*PNLIPRP1*) + 0.0002*log_2_(*CAP2*) + 0.1098*log_2_ (*GNMT*) + 0.0958*log_2_(*MAT1A*)-0.0415*log_2_(*ABAT*) + 0.1113*log_2_(*GPT2*)-0.0352*log_2_(*ADHFE1*)-0.0863*log_2_(*PHGDH*) + 0.0120*log_2_(*PSAT1*)-0.0180*log_2_(*ERP27*)-0.0302*log_2_(*PDIA2*)-0.0999*log_2_(*MT1H*) + 0.0770*log_2_(*COMP*)-0.1238*log_2_(*COL5A2*) + 0.2361*log_2_(*FN1*)-0.0729*log_2_(*COL1A2*)-0.0560*log_2_(*FAP*) + 0.1020*log_2_(*POSTN*), log_2_(gene count). The results were shown in Additional file 2: Figure S2 that none of 19 genes could be used solely for diagnosis in that the AUC of them were almost less than 0.90. The solution paths and parameters of lasso regression model of 19-genes diagnostic risk model were shown in Fig. 5a and b. The distribution of risk score of normal and tumor group of training set-GSE28735 and validation set-GSE16515 were shown in Fig. 5c and e respectively. The results in Fig. 5d and f both indicated the co-detection of these genes that exhibited excellent performance of risk score in diagnosing pancreatic cancer using ROC analysis. The AUC in training set-GSE28735 was 0.975, *P* < 0.0001 and in validation set-GSE16515 was 0.948, *P* < 0.0001. In consideration of accuracy and feasibility, we simplified the diagnostic risk model by removing mRNAs with low weight step by step. As shown in Fig. 5g, as the number of gene decreased, the overall trends of AUC values of training and validation cohort were downward. We set AUC > 0.90 as the cut-off value to get the simplified diagnostic risk model with minimum quantity of gene count. A four-gene diagnostic risk model was built: 0.3034*log_2_(*MAT1A*)-0.1526*log_2_(*MT1H*) + 0.4645*log_2_(*FN1*)-0.2244*log_2_(*FAP*), log_2_(gene count). The AUC in training cohort-GSE28735 of simplified model was 0.954, *P* < 0.0001 (Fig. 5i) and in validation cohort-GSE16515 was 0.928, *P* < 0.0001 (Fig. 5k)*.*

### Construction and validation of prognostic risk model

**Fig. 6:**
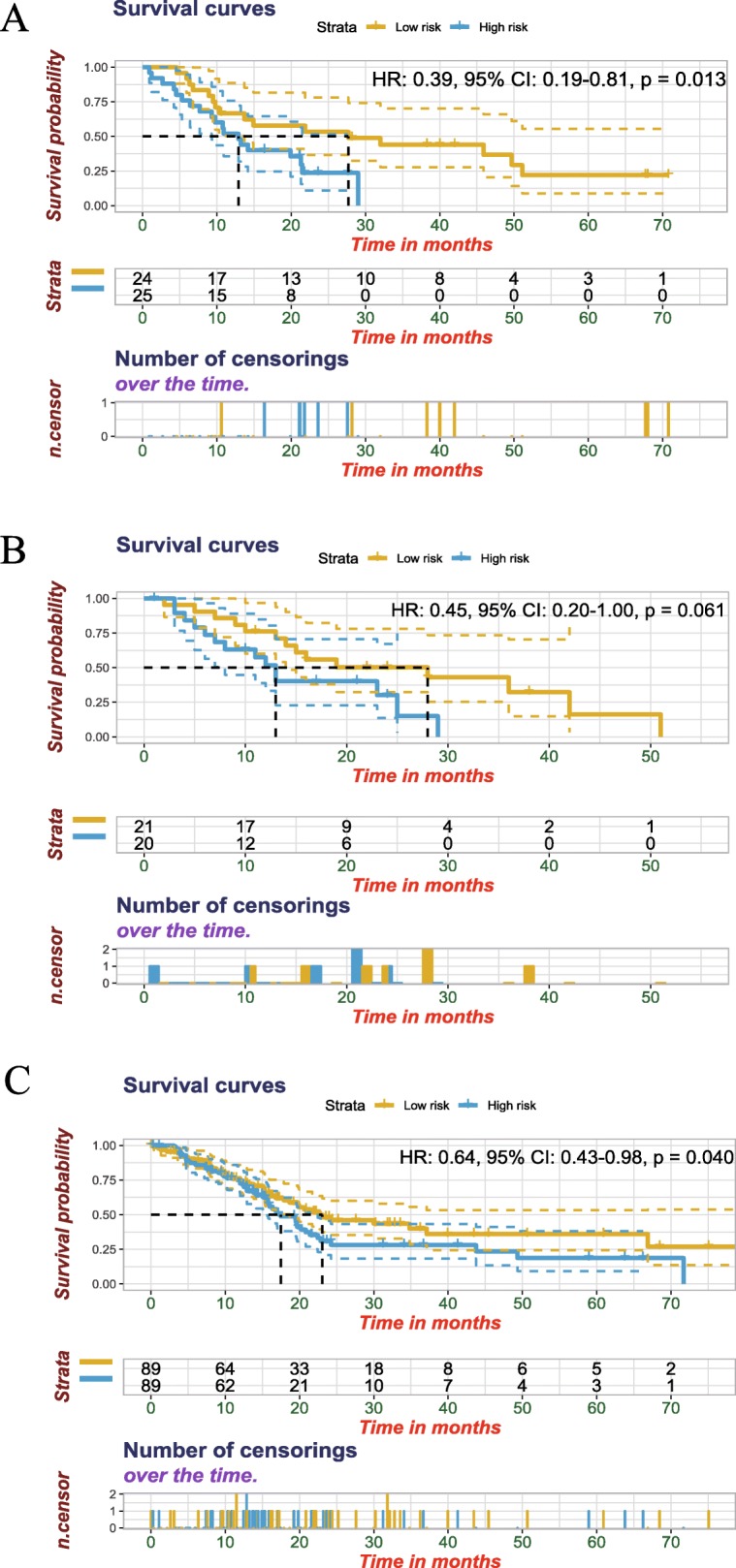
Four-gene prognostic model of pancreatic cancer in training and validation cohort. **a** Construction of prognostic risk model of pancreatic cancer in training cohort-GSE78229. Yellow and blue represent patients with low and high risk scores. We defined high risk group as risk score ≥ median, low risk group as risk score < median. **b** Validation of prognostic risk model of pancreatic cancer in GSE28735. **c** Validation of prognostic risk model of pancreatic cancer in TCGA. TCGA, The Cancer Genome Atlas

To identify the prognosis-associated genes in sub-clusters, prognostic risk model was conducted, which included genes *CEL*, *CPA1*, *POSTN*, and *PM20D1*. The formula for the prognostic risk scores used in this study was as follows: prognostic model = − 1.400*log_2_(*CEL*) + 1.321*log_2_(*CPA1*) + 0.454*log_2_(*POSTN*) + 1.011*log_2_(*PM20D1*). The patients involved in the research were separated into two groups, high-risk group (*N* = 20) and low-risk group (*N* = 21) (Fig. 6a). It was indicated that patients in high-risk group tended to exhibit shorter survival time while in low-risk group, patients had the lower mortality (HR: 0.39, 95%CI: 0.19–0.81, *P* = 0.013). To further test the finding, the risk model in GSE28735 and TCGA was reevaluated. Although in GSE28735 database, it seemed to have no prognostic value for the *P* value = 0.061 (Fig. 6b). The patient samples in the database were small, only 41. To compensate its defects, another validation in TCGA was performed. Similarly to the training cohort, the results in Fig. 6c (HR: 0.64, 95%CI: 0.43–0.98, *P* = 0.040) revealed that the high-risk group had the higher mortality than that in low-risk group. Beside, we compared the prognostic performance between the prognostic model and existing prognosis models. The prognostic risk model we defined had a preferable prognostic performance in both GSE78229 and TCGA cohort (Additional file 3: Figure S3).

## Discussion

Application of the public archives is a powerful weapon to understand three fundamental questions of cancers: from exploring cancer biology, to prediction of progression, and treatments to which it will respond (Rung and Brazma, 2013). However, many difficulties are encountered in data collection, analysis and annotation for the rather noisy data from one individual research. Thus, integrating different databases can generate valuable resources and overcome the rather noise from different individual study (Dai et al., 2017). In this study, we adopted an integrated analysis, the RRA method, to select significant DEGs from four independent datasets of PC gene chips, which could provide more convincing research results.

Ultimately, 138 up-regulated and 165 down-regulated DEGs were selected by RRA methods from the four independent datasets of PC. Part of them have been documented to be tumor promoter genes of PC, such as *GABRP*, *CEACAM5*, *CEACAM6* and *CST1* (Takehara et al., 2007; Govindan et al., 2009; Riley et al., 2009; Jiang et al., 2015). Some of them are considered as the tumor suppressor genes of PC, such as *PLA2G1B*, *SERPINI2* and *NR5A2* (Goonesekere et al., 2018; Bailey et al., 2016; Murtaugh, 2014)*.* Several genes have been proven to be the prognostic or diagnostic biomarkers of PC, such as *LCN2*, *CLDN18*, *LAMC2* and *SULF1*(Bartsch et al., 2018; Ito et al., 2011; Kosanam et al., 2013; Lyu et al., 2018). GO enrichment analysis of up-regulated genes revealed that these significant genes were highly related to extracellular matrix (ECM) regulation, which was consistent with the clinical features of PC: early local invasion/distant metastasis (Wray et al., 2005). Enrichment analysis of down-regulated genes is also indicative of the close connection between these significant genes and another crucial pathway in PC (Halbrook and Lyssiotis, 2017; Michalski et al., 2017). Furthermore, we also discovered many DEGs, whose roles in PC are still ill-defined, such as *IFI27*, *KRT17*, *COMP* and *COL8A1*. Their functions need to be further researched in PC.

Next, WGCNA and co-expression networks were used to identify the hub genes of PC. The significant modules of WGCNA were involved in ECM regulation, metabolism correction pathways, PI3K-Akt signaling pathway and platelet derived growth factor signaling pathway, which have been widely studied in PC (Vaquero et al., 2003; Stoll et al., 2005; Weissmueller et al., 2014). Prognostic and diagnostic predictive models in multiple cancers could be identified according to the information of clinical indicator, pathological classification and related gene expression (van ’t Veer et al., 2002a; van ’t Veer et al., 2002b). Finally, a set of robust prognostic signatures including *SYCN*, *PNLIPRP1*, *CAP2*, *GNMT*, *MAT1A*, *ABAT*, GPT2, *ADHFE1*, *PHGDH*, *PSAT1*, *ERP27*, *PDIA2*, *MT1H*, *COMP*, *COL5A2*, *FN1*, *COL1A2*, *FAP* and *POSTN* were identified by lasso regression analysis from DEGs and could diagnose the PC. Then, a four-gene simplified diagnostic risk model was built. A four-gene prognostic signature composing *CEL*, *CPA1, POSTN* and *PM20D1* was established by Cox proportional hazards regression model combined with Kaplan-Meier survival analysis and could predict the overall survival of PC.

Early diagnosis of PC has always been the challenge in cancer field (Keiji Hanada et al. 2017). *CA19–9* is perhaps the widely evaluated tumor marker in PC patients, while its universal applicability in the diagnosis of PC was severely limited for the non-specific expression in several benign and malignant diseases (Balakrishnan et al., 2007). A great deal of effort has been made for the early detection of PC, and put forward kinds of diagnostic biomarkers for PC, such as ICAM-1, OPG, TIMP-1 (Brand et al., 2011). However, these biomarkers have also not broken through the dilemma of difficult detection of early PC. Endoscopic ultrasound-guided fine needle aspiration (EUS-FNA) is a new development technique for forecasting the quality of pancreatic neoplasm in recent years (Puli et al., 2013). Previous analysis showed that EUS-FNA displayed a high specificity, but lower sensitivity (Puli et al., 2013). Though EUS-FNA has shortcomings in PC diagnosis, it is an efficient method to obtain tissues of pancreatic neoplasm besides surgery. Hence, building an effective diagnostic risk model based on gene detection in pancreatic neoplasm is of great importance in PC diagnosis and may break the state quo. In this study, we firstly confirmed a 19-gene prognostic model through integrated transcriptomic analysis. Among these genes, *SYCN* and *POSTN* have been reported as the diagnostic biomarkers in PC (Makawita et al., 2013; Dong et al., 2018). *PNLIPRP1* and *GNMT* have been documented as the tumor suppressor genes of PC (Goonesekere et al., 2018; Zhang et al., 2013) and *PHGDH* been certified as the tumor promoter gene of PC (Song et al., 2018). This risk model could accurately diagnose PC in our subsequent verification, while many variables in this model need to be controlled, resulting in limited application of these prognostic signatures in clinic in the future. In addition, this model was based on the DEGs from surgical specimen of PC. It remains unclear whether this model displayed an excellent accurate diagnosis in the tissues from EUS-FNA. The expression of these biomarkers should be detected in a large number of tissues from EUS-FNA to confirm the high specificity and sensitivity of the diagnostic model. Thus, further study is necessary to the clinical application of the model for PC diagnosis.

PC is one of the worst prognosis cancers, making the prognostic biomarkers becoming especially important in PC. Recently, it has been reported that several predicted models for risk estimation, such as *S100P*, *ERO1LB*, *SULF1*, *ITGA2*, *GPRC5A, ACTN4, LMO2, p16INK4a* and *CLPS* (Watanabe et al., 2015; Nakata et al., 2009; Gerdes et al., 2002; Lyu et al., 2018; Zhang et al., 2013; Ji et al., 2014; Zhu et al., 2017; Li et al., 2018; Liu et al., 2018). A study recently identified three genes *COL11A1*, *GJB2* and *CTRL* as prognostic biomarkers for PC by using integrated whole genome microarray analysis and immunohistochemical assay (Sun et al., 2018). Most existing prognosis models of PC involve only one gene or mRNA, which have their limitations. Because the expression and crosstalk of multiple genes jointly account for the outcome of PC. That’s the reason why we paid far more attention in identifying co-expression networks and hub genes. The prognostic model that we built involves multi-hub genes that interact in different modules and pathways, which improves the specificity and reliability of the model. Testing of serum miRNAs has been a novel method for predicting the outcome of PC patients. Researchers from Nanjing, PR China identified a six-miRNA (miR-19a-3p, miR-192-5p, miR-19b-3p, let-7b-5p, miR-223-3p, and miR-25-3p) signature in the serum for PC early and noninvasive diagnosis (Zou et al., 2019). Besides, a study was to identify a prognostic model that combined the clinical factors-distance from common hepatic artery or superior mesenteric artery and biomarker *CA19–9* to predict the outcome, which also indicated that one gene or mRNA could not fully forecast the outcome (Suzuki et al., 2019). Anyway, using clinical factors related models for predicting survival of PC are intuitive. Although a mass of clinical prediction models for PC have been reported, most succumb to bias and have not been validated externally (Strijker et al., 2019).

In this paper, we identified a four-gene prognostic signature for PC, containing *CEL*, *CPA1, POSTN* and *PM20D1*. Compared to the study of Defeng Sun et al., we included more quantified datasets of PC. Poor prognosis of PC may be due to the hallmarks of easy migration and resistance. *POSTN* has been reported to be related to the resistance and invasion in cancers (Park et al., 2016; Landré et al., 2016). In PC, periostin, encoded by POSTN, could enhance the invasiveness and resistance ability of PC cells via activation of the PI3 kinase pathway (Baril et al., 2007). CPA1 could promote the development of PC via ER stress (Tamura et al., 2018) and *CEL* has also been reported as the risk factor of PC (Dalva et al., 2017). *PM20D1* is related to the metabolism pathway (Long et al., 2016), and it may be involved in cancer via influencing tumor metabolism (data no shown). These previous documents have also highlighted the potential role of the four genes in PC. Here, the results of survival analysis cross-checked the accuracy of this prognostic risk model in different cohort and indicated that these four genes could serve as predictive biomarkers for PC.

Rapid development of technology platforms, free access to many published experimental datasets and different statistic values account for the diversity of methods to treat the same question. RRA method is a rigorous way to integrate their results in an unbiased manner for getting rid of noise and error (Kolde et al., 2012). The candidate genes were obtained from RRA analysis of four independent gene chips with great statistic difference. The molecular biology experiments discussed above indicated the functional role of predictors in cancers, while there is little experimental evidence to demonstrate their role in PC. Biological systems are holistic and complicated. Bioinformatic findings provide theoretical guide for basic experiments. Biostatistical and bioinformatics approaches to biological systems will definitely require experimental validation to define their biological relevance.

We have to admit some limitations of this study. Firstly, a major issue is that we can’t collect enough cases of PC in our own institute due to the characteristic of PC. Secondly, due to the lack of the details on TNM stage, symptoms, complications, metastasis, treatment, etc., we can’t make sure that the diagnostic risk model could be used in any manner to diagnose PC and whether the biomarkers could be further tested as serum markers for surveillance purposes. But the candidate genes were selected from four independent gene profiles and diagnostic and prognostic risk model were both validated in other cohort, which could make up for it slightly. We have already collected specimen of PC in different centre with adequate information and the finding would be further verified not long in the future.

## Conclusions

Integrated transcriptomic analysis identifies two four-hub gene modules as specific predictive signatures for the diagnosis and prognosis of PC, respectively. Further study of these hub genes may improve the clinic status of pancreatic cancer therapy.

## Supplementary information

**Additional file 1: Figure S1.** Standardization of gene chips.

**Additional file 2: Figure S2.** ROC curve of candidate genes for diagnosing pancreatic carcinoma respectively. Red line represented sensitive curve, blue line represented identify line.

**Additional file 3: Figure S3.** Comparison of prognostic performance of each prognosis models. (A) The Hazard ratios, 95% confidence intervals (CI) of existing prognosis models were calculated by univariate Cox regression in GSE78229(*n* = 49). (B) The univariate Cox regression of existing prognosis models in TCGA(*n* = 179).

## Data Availability

All data generated or analyzed are included in the “Materials and Methods” section.

## Abbreviations

AUC: Area under the curve
BP: Biological process
CC: Cellular component
DEGs: Differentially expressed genes
ECM: Extracellular matrix
GEO: GENE Expression Omnibus
GO: Gene Ontology
KEGG: Kyoto Encyclopedia of Genes and Genomes
Lasso: Least absolute shrinkage and selection operator
MCODE: Molecular complex detection
MF: Molecular function
PC: Pancreatic cancer
PPI: Protein-protein interaction
ROC: Receiver operating characteristic
RP: Reactome pathway
RRA: Robust Rank Aggreg
SAGE: Serial analysis of gene expression
TCGA: Cancer Genome Atlas Cancer Genome
WGCNA: Weighted gene co-expression network analysis
